# A Hybrid θ*-APF-Q Framework for Energy-Aware Path Planning of Unmanned Surface Vehicles Under Wind and Current

**DOI:** 10.3390/s26072116

**Published:** 2026-03-29

**Authors:** Xiaojie Sun, Zhanhong Dong, Xinbo Chen, Lifan Sun, Yanheng An

**Affiliations:** 1 College of Information Engineering, Henan University of Science and Technology, Luoyang 471023, China; 231404010105@stu.haust.edu.cn (Z.D.); 240320040562@stu.haust.edu.cn (X.C.); lifan.sun@gmail.com (L.S.); 221404010301@stu.haust.edu.cn (Y.A.); 2 School of Automation and Intelligent Sensing, Shanghai Jiao Tong University, Shanghai 200240, China; 3 Key Laboratory of Technology and System for Intelligent Ships of Liaoning Province, Dalian Maritime University, Dalian 116026, China

**Keywords:** unmanned surface vehicle, hybrid path planning, reinforcement learning, artificial potential field, energy-aware navigation

## Abstract

Safe and energy-aware navigation is still difficult for unmanned surface vehicles (USVs), especially in cluttered waters where obstacles, smooth motion, and wind or current effects must be considered at the same time. If these issues are handled separately, the path may become longer and the vehicle may turn more often, which raises propulsion effort and hurts stability. To reduce these problems, a hybrid path planning method called $\theta^{*}$-APF-Q is proposed, and it combines global planning, learning-based decisions, and local adjustment in a three-layer structure. First, an any-angle $\theta^{*}$ global planner is employed to generate a near-optimal backbone trajectory by line-of-sight pruning, thereby reducing redundant waypoints and limiting detours. Second, an enhanced tabular Q-learning model is executed in an expanded eight-direction action space, and policy learning is guided by a multi-objective reward that jointly encourages distance reduction, alignment with ocean current and wind-induced forces for energy saving, smooth heading variation to suppress excessive steering, and maintenance of a safety margin near obstacles. Third, an adaptive artificial potential field (APF) module is used for real-time local correction, providing repulsion in high-risk regions and assisting trajectory smoothing to reduce unnecessary turning operations. A decision bias strategy further couples instantaneous APF forces with long-term state–action values, while the influence weight is adaptively adjusted according to environmental complexity. The algorithm is validated on the randomly generated marine grid maps and on the real-world satellite map scenario, with comparisons against a conventional four-direction Q-learning baseline. Across randomized tests, average path length, turning frequency, and the composite energy indicator are reduced by 22.3%, 55.6%, and 26.4%, respectively, and the success rate increases by 16%. The results indicate that integrating global guidance, adaptive learning, and local reactive decision making supports practical, energy-aware USV navigation.

## 1. Introduction

Unmanned surface vehicles (USVs) are transforming autonomous maritime operations and accelerating the evolution of shipborne autonomy and navigation technologies [1]. Owing to their autonomy, cost-effectiveness, and high maneuverability, USVs are increasingly deployed in missions that are hazardous, repetitive, or time-consuming for manned platforms. The USVs are used in tasks such as transport support, sea monitoring and interception, marine patrol, escort missions, and cooperative multi-vehicle operations [2]. In these tasks, the USV often needs to move through crowded waters with reefs, buoys, and docks, and it must also deal with wind and ocean currents. Many USVs rely on batteries or have limited fuel, so the energy used during navigation has a direct effect on mission endurance and operational range. At the same time, repeated turns and oscillatory maneuvers can speed up wear and make it harder to move steadily in narrow areas. For these reasons, a path planner should not only avoid collisions, but also keep the route smooth, energy-aware, and remain stable when the environment changes.

Path planning methodologies for USVs are commonly categorized into global planning and local planning. Global planners aim to compute a complete route from a start state to a goal state under the assumption that the environment map is known. Representative global approaches include evolutionary optimization such as genetic algorithms [3,4], graph-search and shortest-path methods such as Dijkstra [5], and swarm-intelligence methods such as ant colony optimization [6]. For instance, an improved ant colony optimization (ACO) algorithm incorporating fuzzy logic has been proposed for USV local path planning under multi-modality constraints, showing better adaptability in dynamic environments with static and moving obstacles as well as current effects [7]. These methods can produce collision-free routes with favorable optimality properties on static maps; however, they typically rely on accurate prior maps and may be computationally demanding when the environment becomes large or highly cluttered. In contrast, local planning focuses on real-time decision making using local sensing information, and is designed to react to unknown obstacles. Widely used local techniques include sampling-based methods such as rapidly exploring random trees (RRTs) [8] and heuristic search/optimization approaches such as particle swarm optimization (PSO) [9]. While local planners are responsive, they often suffer from local minima, oscillations, and degraded global optimality, especially when the environment contains narrow passages or moving obstacles. Therefore, achieving both global efficiency and local safety remains a key challenge for USV navigation.

Reinforcement learning (RL) has recently emerged as a promising paradigm for navigation and path planning because it enables an agent to learn decision policies directly through interaction and reward feedback [10]. By learning from data rather than relying solely on handcrafted models, RL can potentially cope with uncertain complex constraints, which is attractive for maritime environments where hydrodynamic effects, sensing uncertainty, and changing currents/wind complicate explicit modeling. Nevertheless, conventional RL-based planners still face obstacles to practical deployment. First, learning can be sample-inefficient in high-dimensional state-action spaces, leading to long training time or unstable convergence. Second, many RL formulations optimize sparse objectives and consequently produce trajectories that are jagged or contain excessive steering, which is undesirable for real USVs. Third, RL policies may have difficulty generalizing across environments when the reward design fails to incorporate physical priors such as smoothness or energy usage. Such limitations have been highlighted in dynamic planning scenarios, where an agent must avoid obstacles while maintaining stable performance under changing conditions [11].

A growing body of work has demonstrated the effectiveness of deep RL for maritime and robotic planning tasks. Han et al. formulated maritime search-and-rescue coverage path planning as a Markov decision process and integrated multiple deep Q-network (DQN) variants, including double DQN (DDQN), prioritized experience replay, distributional learning, and noisy networks, achieving improved route quality and search efficiency compared with conventional methods [12,13,14]. In USV navigation, deep and fuzzy RL has also been explored for intelligent multi-objective path planning, where multiple criteria are optimized in a unified learning framework [15]. For resource-aware multi-agent scenarios, Zhang et al. combined deep reinforcement learning with task-specific coupling mechanisms, improving stability and runtime performance in constrained environments [16]. Beyond pure learning-based policies, hybrid approaches have been explored to inject classical planning priors into the learning process. Xu et al. proposed MAPF-DQN by combining a manipulation-compliant artificial potential field (APF) with DQN, where the potential field shapes the reward and a nonlinear Nomoto model is used for trajectory smoothing, resulting in improved planning efficiency and route safety [17]. These studies suggest that combining learning with structured priors can improve convergence and practical path quality.

Despite these advances, two issues remain particularly salient for energy-aware USV planning in dynamic marine environments. First, many existing approaches optimize distance, time, or success rate without explicitly coupling action selection to environmental assistance. In real operations, currents and wind can increase propulsion energy when opposed and reduce it when leveraged; therefore, energy-aware planning should reflect the alignment between the vehicle motion and environmental forces in the optimization objective and the decision policy. Second, purely global planners may produce routes that are optimal in a static sense but fragile in the presence of dynamic obstacles, whereas purely local planners may yield safe but globally suboptimal or oscillatory paths. Learning-based planners can react to uncertainty but may still generate excessive turning, which increases energy consumption and mechanical wear. Consequently, a robust planner should unify global guidance for efficiency, local reactive decision making for safety, and learning adaptability for dynamic conditions, while explicitly considering energy and smoothness.

To address these challenges, this paper proposes a hybrid, energy-aware path planning algorithm $\theta^{*}$-APF-Q, which is built upon Q-learning and organized as a three-layer synergistic framework [18,19]. In the proposed architecture, a $\theta^{*}$ global planner first generates an any-angle reference route by exploiting line-of-sight checks to prune redundant nodes.

This global planner helps shorten the route and mitigates unnecessary turns when compared with grid-constrained search, so it offers a useful baseline for energy conservation [20]. Based on this reference, an improved Q-learning module makes decisions with an eight-direction action set and uses a reward that can adjust to the environment. In this reward, distance reduction, energy-related benefits from moving with wind/current, penalties on sharp steering, and safety constraints are all considered together, so learning is guided toward paths that are both practical and energy-aware. After that, a local adaptive APF module is used to correct the motion in real time when obstacles are close. With dynamic weights and smoothing strategies, this layer reduces shaking and too many steering changes, which helps lower wear and keep the motion stable [21,22].

The main contributions of this paper are summarized as follows:

1. A hybrid framework is built for USV navigation by combining $\theta^{*}$ global planning, Q-learning decision making, and APF-based local reactions, so that global efficiency, local safety, and online adaptation can be handled together.
2. A multi-goal reward is proposed to support energy-aware and smooth paths by using path length, steering smoothness, obstacle clearance, and alignment with wind/current forces.
3. A fusion strategy is introduced to adjust the roles of global guidance and local potential-field responses during learning and execution, which helps training under sparse rewards and improves performance under wind and current.
4. Comprehensive evaluations are carried out on random grid maps and on the real maritime map, and the results show steady gains in path quality, energy-related cost, turning count, and success rate when compared with a conventional Q-learning method.

The rest of this paper is arranged as follows. Section 2 reviews Q-learning and then describes the proposed $\theta^{*}$-APF-Q method, including the full structure and the key steps used for integration and tuning. Section 3 explains the experimental setup and reports the comparison results with analysis. Section 4 gives the conclusions and lists possible directions for future work.

## 2. Hybrid Path Planning Framework

**Figure 1:**
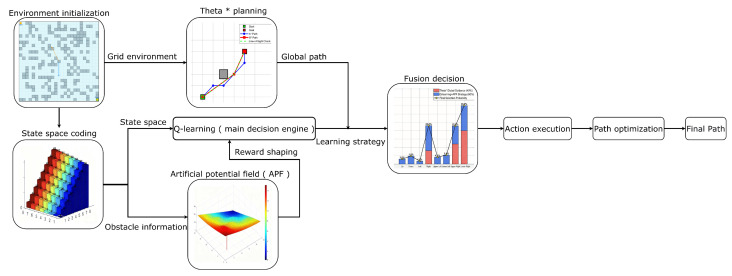
The flow chart of the $\theta^{*}$-APF-Q algorithm.

This section explains the main parts of the proposed $\theta^{*}$-APF-Q framework, which covers the environment modeling, the learning formulation, the global–local coupling mechanism, and the multi-objective optimization strategy. Instead of using the single planners, a three-layer design is established in which an any-angle global planner gives a near-optimal route structure, a model-free learner improves the action choices under changing conditions, and an adaptive potential-field module provides real-time obstacle avoidance and smoother motion. The overall experimental workflow is shown in Figure 1.

### 2.1. Environment Discretization and State Representation

A grid map is used to describe the maritime area so that a finite state space can be built for tabular reinforcement learning [23]. The workspace is divided into a 25×25 grid, and each cell represents a 20m×20m region. Cells occupied by reefs, docks, or other hazards are marked as blocked, and the overall obstacle density is limited so that the task is not always impossible while the scene still remains challenging.

The environment is represented as a composite state

(1) $$E=\{O,E_{\mathrm{ocean}},E_{\mathrm{wind}},P_{\mathrm{current}},P_{\mathrm{target}}\},$$

where *O* is the obstacle set, $E_{\mathrm{ocean}}$ and $E_{\mathrm{wind}}$ describe the ocean current and wind fields, and $P_{\mathrm{current}}$ and $P_{\mathrm{target}}$ are the current and goal positions of the USV. In this way, the original continuous navigation task is turned into a finite Markov decision process (MDP), which makes computation easier and allows the tests to be repeated in a consistent manner.

To allow richer movement choices without increasing the state size, actions are defined in eight directions, including both straight and diagonal moves. Each action *a* is associated with a unit direction vector $v_{a}$ in the grid plane. Collisions are detected by checking whether the next cell belongs to *O*; a terminal success condition is triggered when the target cell m.

### 2.2. Q-Learning Preliminaries

Q-learning is a model-free reinforcement learning algorithm that learns an action policy via trial-and-error interactions without requiring an explicit hydrodynamic model [18,19,24]. A tabular Q-function Q(s,a) is maintained, where *s* is the discrete state and *a* is an action from the eight-direction set. The Q-value represents the expected discounted cumulative reward when action *a* is taken at state *s* and the current policy is followed thereafter.

The Q-table is updated using the temporal-difference form of the Bellman optimality recursion:

(2) $$Q\left(s,a\right)\leftarrow Q\left(s,a\right)+\alpha [r+\gamma \max_{a^{\prime }}Q\left(s^{\prime },a^{\prime }\right)-Q\left(s,a\right)],$$

where α∈[0,1] is the learning rate, γ∈[0,1] is the discount factor, *r* is the immediate reward, and $s^{\prime }$ denotes the successor state. A smaller α is typically selected for stability, while γ controls the extent to which long-term returns are emphasized.

Although standard ϵ-greedy exploration may be employed, purely random exploration is often inefficient in large maritime grids with sparse terminal rewards. Therefore, the exploration process is later shaped by global-path guidance and local-field bias, thereby improving convergence speed and practicality.

### 2.3. Global Guidance via Any-Angle $\theta^{*}$ Search

A global reference path is generated using an any-angle $\theta^{*}$-style planner to reduce detours and unnecessary intermediate waypoints [20]. A core step is the line-of-sight (LOS) test, which checks whether two nodes can be linked by a straight segment without crossing any obstacles. If such a direct link is possible, the parent of the current node can be updated to this better connection. Here, *n* denotes the current node, p(n) is its present parent, and $n_{p}$ represents a neighboring candidate used when the LOS test does not pass. The cumulative cost term g(·) is updated as:

(3) $$g\left(n\right)=\left\{\begin{array}{cc} g\left(p(n)\right)+d\left(p(n),n\right), & \mathrm{LOS}=\mathrm{True},\\ g\left(n_{p}\right)+d\left(n_{p},n\right), & \mathrm{LOS}=\mathrm{False}, \end{array}\right.$$

where d(·,·) denotes the Euclidean distance on the grid (consistent with diagonal moves).

LOS feasibility is tested by a discrete ray-walk on the grid. In practice, the Bresenham line routine is used to check whether a straight segment passes through any blocked cells [25]. When intermediate nodes are removed in this way, the global route becomes closer to a near-shortest geometric path. This route is then used as a reference to guide later learning and to reduce unnecessary exploration in areas that do not help reach the goal.

It should be noted that the global route is used as a soft guide instead of a strict rule. This choice keeps enough freedom during execution, since wind and current fields may change the best local direction even when the precomputed global route is still collision-free.

### 2.4. Adaptive Artificial Potential Field with Wind and Current

For real-time obstacle avoidance and small local adjustments around the global route, an artificial potential field (APF) layer is added as a fast reactive safety module [21,22]. The overall potential is written as the sum of three parts:

(4) $$U_{\mathrm{total}}=U_{\mathrm{att}}+\sum U_{\mathrm{rep}}+U_{\mathrm{env}},$$

where $U_{\mathrm{att}}$ attracts the agent toward the target, $\sum U_{\mathrm{rep}}$ repels it from obstacles, and $U_{\mathrm{env}}$ represents wind and current cues so that motion can follow helpful field directions when possible.

The guidance vector at the current state is obtained from the negative gradient:

(5) $$F_{\mathrm{APF}}=-\nabla U_{\mathrm{total}}.$$

When the vessel moves in a tight area with many obstacles, the repulsive part is weighted more to keep a safe clearance. When the space is open, the attractive and environmental parts are weighted more so that long detours are avoided and favorable currents can be used. With this simple switching of influence, repeated left-right swings can be reduced, and the overall travel can be more stable than with a fixed-parameter APF [21,22].

### 2.5. Multi-Objective Reward Design

To match practical maritime needs, the multi-objective reward is designed to cover several goals at once, including short travel, safe clearance, smooth steering, and field-aware motion. The immediate reward is defined as a weighted sum:

(6) $$R=R_{s}+R_{c}+\beta_{d}\Delta d+\beta_{p}\left(v_{a}\cdot F_{\mathrm{APF}}\right)+\beta_{\theta }\Delta \theta +\beta_{s}d_{m},$$

where $R_{s}$ is the terminal success reward and $R_{c}$ is the collision penalty (activated upon entering obstacle cells). The other terms are set as follows:

- Path efficiency term Δd: Progress toward the goal is computed as the reduction in Euclidean distance to the goal between consecutive steps, $\Delta d=d_{\mathrm{curr}}-d_{\mathrm{next}},$ where $d_{\mathrm{curr}}$ and $d_{\mathrm{next}}$ are the distances from the current and next positions to the goal, respectively (in meters).
- Environmental alignment term $v_{a}\;\cdot \;F_{\mathrm{APF}}$: The dot product between the unit direction vector of the chosen action $v_{a}$ and the total potential force $F_{\mathrm{APF}}$ (computed from Equations (4) and (5)) is used as a simple indicator of whether the action follows helpful wind/current directions. With a positive $\beta_{p}$, actions aligned with favorable field cues are encouraged.
- Smooth steering term Δθ: The heading change between two consecutive actions is computed as the absolute angular difference between the direction vectors of the previous and current actions (in degrees). This term penalizes sharp turns to support smoother motion and reduce frequent control changes.
- Clearance term $d_{m}$: The minimum Euclidean distance from the next position to the nearest obstacle is measured in meters (based on the grid cell size of 20m). This term promotes safer margins, and the weight $\beta_{s}$ controls how conservative the policy becomes. The reward expression in Equation (6) is written in separate parts so that each objective is explicit; the overall effect depends on the selected weights $\beta_{d},\beta_{p},\beta_{\theta },\beta_{s}$ and on consistent definitions of Δd and Δθ. By adding dense feedback beyond terminal rewards, learning can be accelerated on large grids, where sparse rewards often slow down tabular methods [18,19].

The current framework represents hydrodynamics and maneuverability in a simplified form. The environmental alignment term in Equation (6) captures wind and ocean current effects by encouraging actions that follow favorable field directions, while the smooth steering term penalizes abrupt heading changes to reflect the limited turning capability of USVs. A full hydrodynamic model is not incorporated; instead, these effects are approximated at the planning level to keep the state space tractable for tabular Q-learning.

### 2.6. Action Biasing for Global–Local Fusion

To connect the three layers, an action-biasing scheme is used. First, a guidance probability shapes exploration along the global path. Next, an energy-based action distribution combines the APF direction cue with the learned *Q* values.

#### 2.6.1. Planning Guidance Probability

A guidance probability $P_{\mathrm{guide}}$ is defined using the angle difference Δθ between the current heading and the local direction suggested by the global $\theta^{*}$ path. A simple exponential form is used:

(7) $$P_{\mathrm{guide}}=0.6\cdot \exp\;\left(-\frac{\Delta \theta }{\sigma }\right),$$

where σ>0 controls how quickly the probability drops as the angle grows. If Δθ is small, $P_{\mathrm{guide}}$ becomes larger and actions close to the global path are more likely. If the angle is large, the bias becomes weaker, so the policy can still react to obstacles and field changes.

This setup also supports a simple learning schedule. At the beginning, the bias can reduce wandering by keeping the agent near a useful areas. Later, as the *Q* values become stable, more weight can be placed on learned experience and local cues.

#### 2.6.2. Biased Action Distribution

To mix short-term safety with long-term return, an energy-based sampling rule is used for action selection:

(8) $$\pi \left(a|s\right)\propto \exp\;\left(\beta \;\left(v_{a}\cdot F_{\mathrm{APF}}\right)+Q\left(s,a\right)\right),$$

where $F_{\mathrm{APF}}$ is computed from Equations (4) and (5), and $v_{a}$ is the unit vector of action *a*. The factor β is changed with local complexity: it is increased near dense obstacles so that collision avoidance is stressed, and it is reduced in open areas so that long-term value learning plays a larger role.

With this design, the policy changes with the scene. Close to obstacles, APF repulsion has a stronger effect. In free space, the *Q* values and the global path bias favor smoother and shorter motion with better use of wind/current.

#### 2.6.3. Multi-Objective Decomposition and Coupling

The framework can be viewed as combining three types of guidance in one decision process:

1. Global path optimality: The LOS-pruned $\theta^{*}$ path gives a long-range direction, and Equation (7) turns this information into a soft bias.
2. Local reaction: The APF layer gives fast corrections through Equations (4) and (5), which helps keep safe distances and follow helpful field directions.
3. Long-term value: Q-learning stores delayed effects in Q(s,a) via Equation (2), which supports better choices over many steps.

The three parts are linked by Equation (8), where $v_{a}\cdot F_{\mathrm{APF}}$ gives a local direction preference and Q(s,a) gives the learned evaluation. At the same time, Equation (7) changes which states are visited more often by keeping exploration near the global path. This joint design helps avoid common problems of single methods: pure global planning may ignore local field changes, while pure potential fields may fall into local minima [22].

#### 2.6.4. Mitigation of Sparse Rewards and Improved Learning Efficiency

In large maritime grids, using only terminal rewards often leads to slow learning. The reward in Equation (6) adds step-by-step feedback using distance progress, clearance, turning smoothness, and environmental alignment. Together with the path bias in Equation (7), exploration becomes more guided and less random, which can reduce wasted samples and speed up convergence [18,19].

The learning process converges steadily under the proposed design. The ε-greedy exploration rate decays linearly over episodes, shifting the policy from exploration to exploitation. The dense reward in Equation (6) provides feedback at every step, avoiding the slow convergence often caused by sparse terminal rewards. Under the same training budget, the enhanced algorithm achieves a higher success rate and often terminates earlier after consecutive successful episodes. By comparison, the baseline Q-learning typically requires more episodes to reach a stable policy because it does not use global guidance and provides weaker step-wise reward signals.

#### 2.6.5. Computational Characteristics and Real-Time Feasibility

The runtime cost is shared across modules. The $\theta^{*}$ path is computed once for a map (or updated only when needed), and it limits the area where heavy exploration is required. The APF layer runs at each step with low cost because it only needs local gradient calculations. The Q-learning update is also light since it is a table update. This split supports real-time use while keeping the decision quality at a practical level [20,21].

A brief complexity analysis further supports the real-time feasibility of the proposed framework. The $\theta^{*}$ global planner is invoked once per map (or only when the environment changes). Its core operation is an $A^{*}$-like search on the grid with additional line-of-sight checks, and the worst-case complexity is O(NlogN), where N=25×25=625 is the number of grid cells, which is negligible for an offline pre-computation step. At each decision step, the Q-learning update only requires accessing and updating a single entry in the Q-table, which is O(1). The APF module computes distances to obstacles within the repulsion range; in the worst case, it scans the entire obstacle set with complexity O(K), where K≤0.35N≈219. This per-step cost is low and scales linearly with the number of obstacles. Since the three modules are only lightly coupled, the global guidance can be computed offline, the learning step updates a small Q-table, and the local correction uses simple geometric calculations; therefore, the overall computation remains low and is suitable for real-time use on typical embedded hardware.

### 2.7. Algorithm Summary

For clarity and repeatability, the full procedure is listed below:

1. Initialization: The grid map is constructed, and wind/current fields are set. The *Q* table entries are initialized, and safety parameters and reward weights are specified.
2. Global planning: A LOS-enabled $\theta^{*}$ path is generated using Bresenham checks.
3. Learning and navigation loop: At each step, APF forces are computed (Equations (4) and (5)), the guidance probability is obtained (Equation (7)), and an action is sampled using the biased policy (Equation (8)). The reward is evaluated (Equation (6)), and the *Q* table is updated (Equation (2)).
4. Termination: The process stops when the target is reached or a collision occurs, and the learned policy is stored for subsequent execution.

With these steps, global guidance, field-aware learning, and local reactive safety are combined into one method for USV navigation under wind and current effects.

## 3. Experimental Evaluation and Performance Analysis

This section reports the experimental tests and analyzes the performance of the proposed $\theta^{*}$-APF-Q planner against a standard tabular Q-learning method. The evaluation focuses on several important measures. Navigation efficiency is measured by the final path length and by how often the route changes direction. A composite energy proxy is also used to reflect the cost of long travel and frequent steering. In addition, the required training time is recorded to show the computing load. Finally, robustness is assessed by the success rate, defined as the percentage of trials that reach the goal without collision under obstacle constraints. In addition to quantitative averages, representative trajectories are examined to clarify how the proposed fusion strategy affects route geometry and steering behavior.

### 3.1. Experimental Instructions

A systematic evaluation framework was constructed to ensure that both planners were assessed under identical environment realizations and initial/terminal conditions. The baseline decision making was implemented as a conventional model-free Q-learning process based on trial-and-error updates of a discrete state–action value table. The hybrid decision making combined an any-angle global guidance module using $\theta^{*}$ with line-of-sight (LOS) pruning; a local reactive obstacle-avoidance module based on an improved artificial potential field (APF); and an integrated decision and training loop in which global guidance and local potential information bias action selection. LOS feasibility checks were performed using a Bresenham-type rasterization strategy to determine whether a direct segment could be connected without intersecting obstacle cells. The use of a discretized grid map allows direct computation of collision constraints and successor transitions and follows common practice in obstacle-grid planning benchmarks.

For the primary benchmark, 400 maritime maps were generated with a fixed grid resolution of 25×25. Obstacle density was controlled below 35% to balance environmental complexity and path feasibility. The grid resolution (25×25) represents a compromise between navigation accuracy and a state-space size that remains manageable for tabular Q-learning. A coarser grid (e.g., 15×15) would reduce the number of states but may oversimplify obstacle shapes and narrow passages, which can affect feasibility. In contrast, a finer grid (e.g., 50×50) would improve spatial accuracy but increases the state space quadratically, making tabular methods impractical. The modular design of the proposed framework helps address this scalability issue: the $\theta^{*}$ planner and the APF module are largely resolution-agnostic and can be applied at different grid scales, while the learning component can be upgraded independently. For example, replacing tabular Q-learning with a function approximator such as a deep Q-network (DQN) would allow scaling to higher resolutions without changing the global-planning or local-reaction modules. This separation makes the framework adaptable to different resolution requirements in real-world deployments.

Wind and current fields are generated once for each environment instance using a uniform random distribution. The wind magnitude is sampled from [0.5,1.8] (dimensionless scale) and the wind direction is sampled from [0°, 360°]; the ocean current magnitude and direction follow the same ranges. For a given map instance, the fields remain static throughout training and navigation, i.e., they do not change within an episode or between steps. Both the baseline Q-learning and the proposed hybrid method are evaluated under exactly the same field realizations for each map, ensuring a fair comparison.

The start and goal locations were fixed to the upper-left and lower-right grid cells, respectively, in order to standardize difficulty across map realizations and to facilitate direct averaging across trials. Wind and current fields were assigned as vector quantities to the grid, and their direction and intensity were visualized using arrow glyphs in the trajectory plots. In implementation, three functional modules were employed: an environment initialization module that generated randomized grids or imported real-world images for grid conversion; an algorithm execution module that performed training and navigation using either the baseline or hybrid method; and a visualization and evaluation module that plotted trajectories and automatically computed the performance indices described below. Both methods were tested on the same set of maps with the same start/goal pair and the same stop rules, so that any gap in results comes from the algorithms rather than from different scenarios.

To check whether the method also works in a more realistic setting, a satellite image of the Zhoushan Islands was transformed into a grid map with the same 25×25 resolution. This map keeps the main geographic layout and still fits the discrete action space used by tabular Q-learning and the grid-based hybrid planner. Since the coarse grid can miss small obstacles and may change the shape of narrow channels, this test is treated as an approximate version of the real area. Even so, it offers a clear and meaningful case for comparing path smoothness, detours around obstacle groups, and overall computing cost.

For each run, four basic measures were recorded: the path length *L* (m), obtained by adding the lengths of all segments on the final route; the number of inflection points $N_{\mathrm{turn}}$, counted as the number of heading changes along the route; the training time $T_{\mathrm{train}}$ (s), measured as the total time used for learning under the same stop rules and settings; and the success rate *S* (%), defined as the share of trials that reach the goal without collision. In addition, the proxy energy value was used to combine travel distance and steering activity. As reported in the result tables, it is computed by

(9) $$E_{\mathrm{proxy}}=0.1\;L+0.5\;N_{\mathrm{turn}},$$

where the weights follow the same rule used in the tables. While $E_{\mathrm{proxy}}$ is not a full power model, it gives a simple and useful index of effort in grid navigation: longer paths usually need more propulsion, and more turns mean more control actions and possible speed changes.

The coefficients are selected such that one turn corresponds to an equivalent energy cost of 5 of straight travel (0.5/0.1=5), reflecting a typical trade-off between steering and propulsion for small USVs. The relative ranking of the compared algorithms remains unchanged when this ratio is varied within a reasonable range (e.g., 2.5–10m per turn), indicating that the proxy energy provides a consistent basis for comparison. The same formula is applied consistently across all randomized maps and the real-world scenario, and the relative ranking of the compared algorithms remains unchanged across these cases, supporting its validity as a unified comparative metric within the scope of this study.

The main parameters used in the experiments are summarized in Table 1. The baseline Q-learning and the proposed enhanced algorithm ($\theta^{*}$-APF-Q) are configured with distinct parameter sets to reflect their respective complexity and design goals.

The environment is discretized into a 25×25 grid with a cell size of 20m. The obstacle density is limited to ≤35%. Wind and current magnitudes are uniformly sampled from [0.5,1.8], and their directions are randomly assigned. And a linear exploration decay is used for both algorithms:

(10) $$\varepsilon =\max\;\left(\varepsilon_{\min},\;\varepsilon_{0}\left(1-\frac{\mathrm{episode}}{1.5\times \mathrm{total\_episodes}}\right)\right).$$

For the enhanced algorithm, a global-path guidance probability σ is applied: with probability σ, the action pointing toward the next waypoint on the $\theta^{*}$ global path is selected; otherwise, the standard ε-greedy rule is used. This implementation corresponds to the guidance mechanism in Equation (7). The parameter β in Equation (8) is not implemented as a separate term; instead, the global-path bias is directly enforced through the probability σ. For the APF force composition, attractive and repulsive forces are computed separately and then combined with fixed weights (attraction weight 1.5 and repulsion weight 0.8) to form the total potential force. This total force is used only in the APF alignment reward term eq:reward Equation (6) and does not directly affect action sampling. Regarding parameter selection, the baseline Q-learning uses a smaller learning rate and a lower exploration level to maintain stable learning with a limited action space, whereas the enhanced method adopts a larger learning rate and a higher initial exploration level to better exploit the richer action space and the added global guidance. All parameters are kept fixed across different maps and environmental conditions to ensure a consistent comparison.

### 3.2. Randomized-Map Performance

The average results over 400 randomly generated maps are summarized in Table 2. When compared with the baseline Q-learning planner, the proposed $\theta^{*}$-APF-Q approach produces shorter routes on average. The mean path length drops from 984m to 764.9m, which is a reduction of 219.1m (22.3%). This result suggests that adding any-angle global guidance helps avoid extra detours that often appear when decisions are driven only by local exploration, even though both methods use the same discretized state representation.

A substantial reduction in maneuvering intensity was also observed. The turning behavior also improves clearly. The average number of inflection points drops from 27 to 12, which corresponds to a 55.6% reduction. This matters in practice because many course changes usually mean more control actions, more wear on steering and propulsion parts, and less stable sensing and communication. The smaller turning count shows that the hybrid method does more than shorten the path; it also produces longer straight segments that better match common USV motion patterns.

A similar trend is seen in the proxy energy value. $E_{\mathrm{proxy}}$ decreases from 112.2 to 82.6 (26.4% reduction). Since this index penalizes both long distance and frequent turning, the drop reflects gains from both sources: the vessel travels less, and it also changes heading less often. Training time is reduced as well, from 6.5s to 4.9s. This result is in line with the use of $\theta^{*}$ guidance [20], which limits aimless exploration, and with the APF term [21,22], which pushes the agent away from unsafe moves and reduces wasted trials.

Robustness is further supported by the success rate. The baseline reaches the goal in 79% of the trials, while $\theta^{*}$-APF-Q reaches 95%, giving a 16 percentage-point increase. This is consistent with the reactive nature of APF-based avoidance [21,22], where repulsive cues near obstacles help the agent keep clearance and avoid getting stuck in dead-end areas.

**Figure 2:**
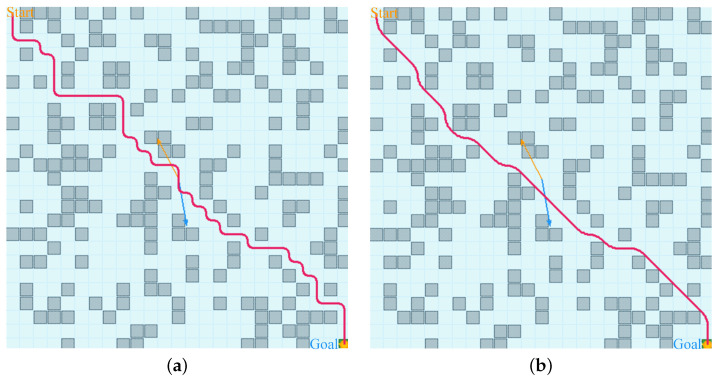
Trajectory comparison on a representative randomly generated map. (**a**) Conventional Q-learning; (**b**); θ*-APF-Q.

A visual comparison is shown in Figure 2. In these plots, gray cells are obstacles and blue cells are free water. Wind and current are displayed by yellow and blue arrows, and longer arrows indicate stronger fields. The baseline Q-learning routes often show zig-zag motion and many small corrections, especially near obstacle groups. In contrast, the $\theta^{*}$-APF-Q routes stay closer to a clear global path and use local changes mainly when they are needed to pass obstacles safely. As a result, unnecessary steering operations are reduced and the route appears more natural. In realistic maritime operations, this type of geometric smoothing is expected to improve endurance and operational efficiency, because fewer and gentler turns generally reduce control activity and mitigate energy losses associated with repeated heading changes.

### 3.3. Real-World Maritime Validation

**Figure 3:**
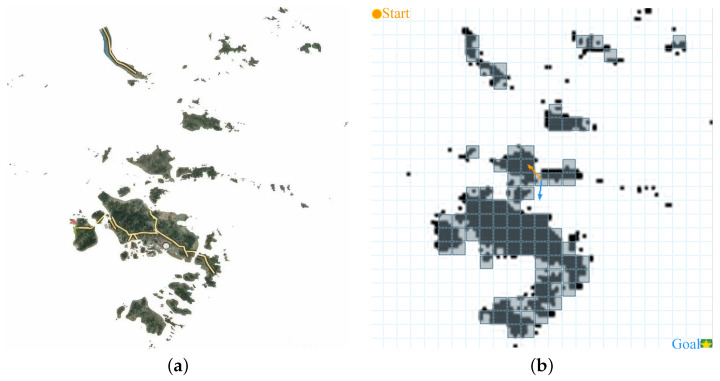
Real-world benchmark from the Zhoushan Islands. (**a**) Original satellite image; (**b**) Converted grid map.

The real-world benchmark was designed to test whether the trends observed on synthetic maps persist when major geographic structures are present. The satellite image and its converted grid representation are shown in Figure 3. Because the map was rasterized to a 25×25 grid, some fine-scale obstacles may be omitted and certain narrow channels may be distorted. Therefore, the real-world study is interpreted as a discretized proxy of the actual geography rather than a high-resolution nautical chart evaluation. Importantly, identical discretization and conversion settings were applied to both planners, so the comparison remains controlled.

Quantitative results are summarized in Table 3. The $\theta^{*}$-APF-Q planner reduced the path length from 960m to 742.25m, giving a reduction of 217.75m. The turning count drops even more strongly, from 26 inflection points to 5, which means the planned path is much smoother and contains longer straight parts with fewer small corrections. In line with these changes, the proxy energy value falls from 110 to 76.73, which matches the joint drop in travel distance and steering frequency. The required training time is reduced from 1.9s to 1.0s, showing that the fusion design keeps the method practical even on a map with real geographic structure.

**Figure 4:**
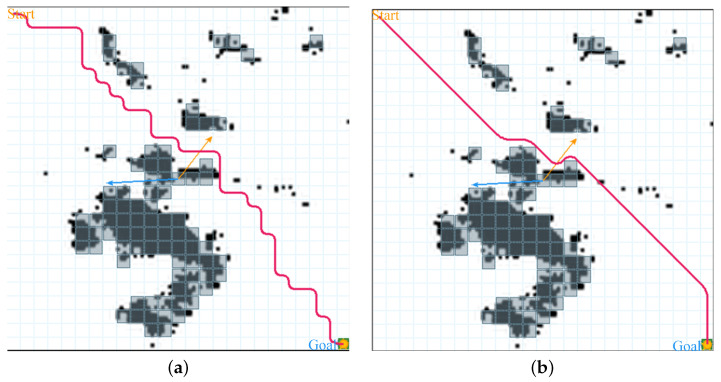
Trajectory comparison on the real-world map. (**a**) Conventional Q-learning; (**b**) θ*-APF-Q.

The planned trajectories for this scenario are visualized in Figure 4. The baseline planner often shows small back-and-forth motions and extra detours when it passes obstacle groups, which increases both the total distance and the amount of steering. By comparison, the hybrid planner usually stays within a clearer global corridor and uses local repulsion mainly when it is needed to keep safe clearance. As a result, the route is closer to common USV behavior, where steady headings and gentle changes are preferred. The lower turning rate is also important in tight waterways, because too many course changes can raise the chance of collision and leave less room for control.

Finally, the limits of map discretization should also be noted. With a coarse grid, the converted map may not fully match the real scene, which can affect the absolute accuracy of the planned route and the shown safety margin. Small objects may disappear, and narrow channels may look wider or may even vanish, depending on the threshold used in the conversion. Even with these limits, the two methods were tested under the same discretization, and the hybrid planner shows stable gains in efficiency, smoothness, robustness, and computing cost when obstacles and environmental effects are present.

## 4. Conclusions

An energy-aware hybrid planning framework for USV navigation in complex marine environments has been developed through the integration of $\theta^{*}$ global guidance, enhanced Q-learning, and an adaptive APF-based local decision-maker. The fusion architecture was shown to alleviate the principal limitations of single-method planners. Detours were reduced and route coherence was improved by $\theta^{*}$ guidance via line-of-sight pruning, whereas collision avoidance and fine-scale adjustments near obstacle boundaries were improved by APF-based reactive correction. In addition, environment-aware decision making was enabled by the learning layer through an expanded action space and a multi-objective reward design in which path elongation and abrupt steering were penalized while motion aligned with beneficial currents and wind was rewarded. Consistent improvements were observed across randomized maps and a real-world scenario: shorter routes, fewer turning maneuvers, reduced composite energy consumption, and higher success rates were obtained relative to the conventional Q-learning baseline, and the resulting trajectories were better aligned with practical USV navigation characteristics. In future work, the framework is expected to be extended toward higher-resolution mapping and continuous-state decision making with realistic dynamic constraints, while physically grounded energy and wear models will be incorporated for improved fidelity. Additional validation under harsher sea states and dynamic obstacle conditions, as well as extensions to multi-USV cooperative planning, is also of interest to enhance reliability and economic viability in real deployments.

## Figures and Tables

**Table 1:** Key experimental parameters.

Parameter	Baseline Q-Learning	Enhanced ($\theta^{*}$-APF-Q)
Learning rate α	0.1	0.3
Discount factor γ	0.8	0.95
Exploration rate ε (initial/min)	0.7/0.05	0.9/0.1
Training episodes	1000	2000
Max steps per episode	500	1000
Attraction gain $k_{\mathrm{att}}$	–	3.0
Repulsion range $d_{\mathrm{rep}}$	–	4 cells (80 m)
Global guidance probability σ	–	0.4
Number of actions	4 (cardinal)	8 (cardinal + diagonal)
Goal/collision/step	+200/−100/−0.2	+200/−100/−0.2
Distance reward coefficient	3.0	3.0
APF alignment coefficient	–	0.6
Turning penalty coefficient	–	0.2

**Table 2:** Average performance on randomly generated maps.

Performance Index	Q-Learning	$\theta^{*}$-APF-Q
Average path length *L* (m)	984	764.9
Inflection points $N_{\mathrm{turn}}$	27	12
Proxy energy $E_{\mathrm{proxy}}$	112.2	82.6
Training time $T_{\mathrm{train}}$ (s)	6.5	4.9
Success rate *S* (%)	79	95

**Table 3:** Performance on the real-world map (Zhoushan Islands) after grid conversion.

Performance Index	Q-Learning	$\theta^{*}$-APF-Q
Path length *L* (m)	960	742.25
Inflection points $N_{\mathrm{turn}}$	26	5
Proxy energy $E_{\mathrm{proxy}}$	110	76.73
Training time $T_{\mathrm{train}}$ (s)	1.9	1.0

## Data Availability

The original contributions presented in this study are included in the article. Further inquiries can be directed to the corresponding author.
